# Lower incidence of diabetes mellitus in patients with aneurysmal subarachnoid hemorrhage: a large case–control study with propensity score matching

**DOI:** 10.3389/fneur.2023.1282486

**Published:** 2023-11-27

**Authors:** Weiying Zhong, Kai Chen, Ziyin Song, Yizhou Xiao, Donglin Zhou, Mingxiang Zhang, Yunyan Wang, Donghai Wang, Wandong Su

**Affiliations:** ^1^Department of Neurosurgery, Qilu Hospital of Shandong University and Institute of Brain and Brain-Inspired Science, Shandong University, Jinan, China; ^2^Key Laboratory of Cardiovascular Remodeling and Function Research, Chinese Ministry of Education, Chinese Ministry of Health and Chinese Academy of Medical Sciences, Qilu Hospital of Shandong University, Jinan, China; ^3^State Key Laboratory of Generic Manufacture Technology of Traditional Chinese Medicine, Lunan Pharmaceutical Group Co., Ltd., Linyi, China; ^4^School of Basic Medical Sciences, Shandong University, Jinan, China

**Keywords:** intracranial aneurysms, subarachnoid hemorrhage, risk factors, diabetes mellitus, propensity score matching

## Abstract

**Background and purpose:**

Diabetes mellitus (DM) is a well-established cardiovascular risk factor for atherosclerotic disease; however, its effect on the risk of rupture of intracranial aneurysms remains controversial. Herein, we aimed to perform a case–control study to investigate the relationship between DM and aneurysmal subarachnoid hemorrhage (aSAH).

**Methods:**

We retrospectively reviewed the data of patients with ruptured or unruptured aneurysms who were treated between 2013 and 2023. Univariate and multivariate analyses were performed. Propensity score matching (PSM) analysis was conducted to evaluate the relationship between DM and risk of aSAH.

**Results:**

A total of 4,787 patients with 5,768 intracranial aneurysms were included. Among them, 2,957 (61.8%) were females, 1765 (36.9%) had ruptured aneurysms, and 531 (11.1%) presented with DM. Female sex, current drinking, and hypercholesterolemia were associated with a higher risk of aSAH, whereas old age, former smoking, and DM were associated with a lower risk of aSAH in multivariate analysis (*p* < 0.05). The incidence of DM (13.4%, 406/3022) in the unruptured group was higher than that in the ruptured group (7.1%, 125/1765) (odds ratio, 0.55; 95% confidence interval, 0.444–0.680) (*p* < 0.001). After propensity score matching, 530 patients with DM were successfully matched, and DM was still associated with a lower risk of aSAH (odds ratio, 0.24; 95% confidence interval, 0.185–0.313) (*p* < 0.001).

**Conclusion:**

Patients with aSAH have a lower incidence of DM, however, this case-cohort study could not establish a causal relationship. A prospective and large study with long-term follow-up is warranted to establish a causal relationship.

## 1 Introduction

Subarachnoid hemorrhage (SAH) has an incidence rate of 9 per 100,000 person-years and accounts for approximately 5% of all strokes (1). Nearly 85% of spontaneous SAHs are caused by ruptured intracranial aneurysms (IA) (2). Despite recent advances in medical and surgical treatment, aneurysmal subarachnoid hemorrhage (aSAH) remains an emergency life-threatening cerebrovascular event; up to 40% of patients with SAH die, and 50–66% suffer permanent disability (3). Even survivors with a “good recovery” may present with memory and neurocognitive impairments and cannot return to work (4). With the widespread use of advanced imaging techniques, an increasing number of unruptured IAs have been incidentally discovered. Therefore, identification of the risk factors for IA rupture and prevention of modifiable risk factors are important. The incidence of aSAH is high in Finland and Japan; it increases with age and is common in women (1). Hypertension, smoking, and alcohol abuse are well-established modifiable risk factors for aSAH, and high total serum cholesterol levels and diabetes mellitus (DM) seem to be inversely associated with aSAH (5, 6). However, not all studies reported an inverse association between DM and aSAH (7–9). Previous studies, particularly population-based cohort studies, often included a small number of aSAH cases. Several studies included all spontaneous SAH cases, regardless of the etiology, or SAH cases with unconfirmed aneurysms (10). The control cases in some cohort studies were generally healthy populations without aneurysms rather than patients with unruptured IAs (7–10). Aneurysm formation and rupture are associated with various risk factors and pathophysiologic mechanisms. Furthermore, not all studies balanced the main confounders of aSAH, such as body mass index (BMI) and cholesterol levels, and different studies may have had different adjusted confounders. Chinese people may have different lifestyle habits from those in the West, and the effect of DM on the occurrence of aSAH in Chinese patients has not been well investigated and may differ from that in other parts of the world. Herein, we aimed to perform a case–control study involving a large sample of patients with ruptured and unruptured aneurysms to investigate the relationship between DM and aSAH.

## 2 Materials and methods

### 2.1 Patients

**Figure 1:**
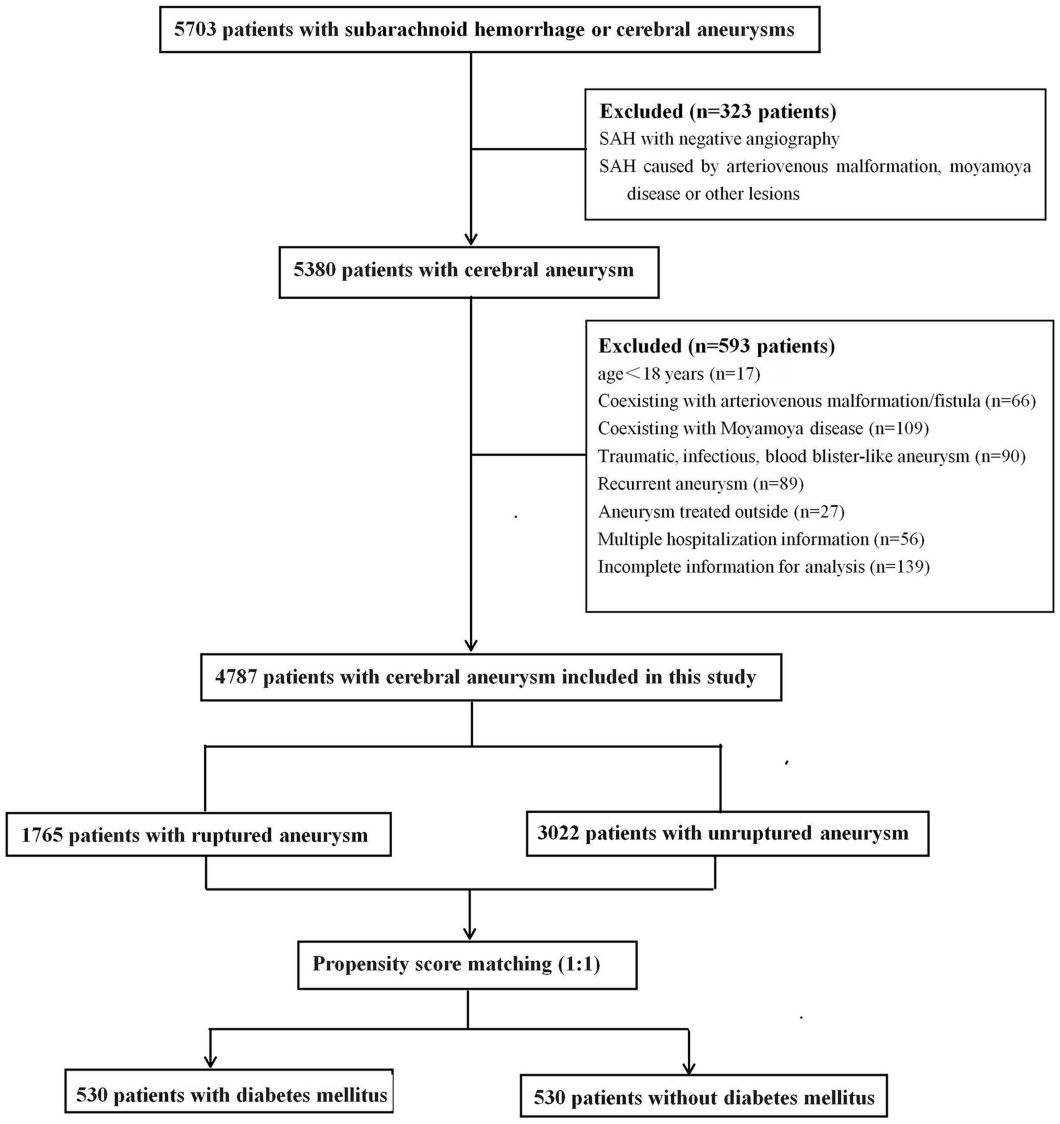
Flow chart of the study population.

This retrospective study was approved by the Ethics Committee of Qilu Hospital of Shandong University, and the requirement for informed consent was waived. From September 2013 to March 2023, 5,703 consecutive patients diagnosed with SAH or cerebral aneurysms were treated at our tertiary hospital and identified in our prospectively collected data. All IAs were confirmed using computed tomography, magnetic resonance imaging, or digital subtraction angiography. SAH was confirmed using computed tomography or cerebrospinal fluid analysis. For patients who received multiple hospitalization treatments at our hospital, only the initial admission information was collected. The exclusion criteria were as follows: (1) age < 18 years; (2) SAH with negative angiography or SAH caused by trauma, arteriovenous malformation, Moyamoya disease, or other structural lesions; (3) aneurysms related to arteriovenous malformation/fistula or Moyamoya disease; (4) traumatic, infectious, or blood blister-like aneurysm; (5) patients who received aneurysm treatment outside of admission; (6) patients with multiple hospitalizations; and (7) incomplete information for analysis. Based on aneurysm status, the patients were separated into ruptured and non-ruptured groups. Patients with multiple aneurysms were assigned to the ruptured group if they presented with SAH. Finally, 4,787 patients with ruptured or unruptured aneurysms were included in the study (Figure 1).

The following information was collected from all participants: demographic characteristics (sex, age, weight, and height), behavioral history (cigarette smoking and alcohol consumption), comorbidities (diabetes, hypertension, and dyslipidemia), and aneurysm status (ruptured or unruptured). BMI was calculated as weight (in kilograms) divided by the square of height (in meters). BMI was divided into the following four groups: underweight (≤18.5 kg/m^2^), normal weight (18.5–24 kg/m^2^), overweight (24–28 kg/m^2^), and obese (>28 kg/m^2^). The cigarette smoking status of the patients was divided into never, former, and current smokers (at least five cigarettes per day). Former smokers were defined as those who had smoked at least five cigarettes per day and quit smoking for 3 months. Alcohol consumption status was categorized as never drinker, former drinker, and current drinker (≥150 g of alcohol per week). Former drinkers were defined as those who drank ≥150 g of alcohol per week and had quit smoking for 3 months. DM was defined as treatment with antidiabetic medication or a fasting glucose level of ≥7 mmol/L during a previous health examination. Hypertension was defined as a history of the disorder (a systolic blood pressure ≥ 140 mm Hg or diastolic blood pressure ≥90 mm Hg), regardless of treatment with antihypertension medication. Fasting serum lipid levels were measured on admission. Hyperlipidemia was defined as patients with a history of dyslipidemia treated with lipid-lowering drugs, a fasting plasma cholesterol level of ≥6 mmol/L, a fasting plasma triglyceride level of ≥2 mmol/L, or a fasting plasma low-density lipoprotein level of ≥3.5 mmol/L. Hypercholesterolemia was defined as a serum total cholesterol level of ≥6 mmol/L. In the analyses, we used the normal-weight, never smoker, and never drinker groups as references. Hypertension, DM, and hyperlipidemia were recorded as present or absent. The patients’ age was divided into ≤50, 50–60, 60–70 and > 70 years, and ≤ 50 years old group was the reference.

### 2.2 Statistical analysis

SPSS (version 23.0; IBM Corp, Armonk, New York, USA) was used for statistical analysis. Continuous variables are presented as means and SD, and categorical variables as numbers (frequency). Continuous variables, such as age and BMI, were transformed into categorical variables, as aforementioned. Fisher’s exact test or Pearson *χ*^2^ test was used to determine predictive factors for aSAH. Factors with *p* < 0.1 in the univariate analysis were considered potential independent variables and subsequently included in the binary logistic regression analysis using an enter process to determine the independent predictors. Statistical significance was set at *p* < 0.05.

Propensity score matching (PSM) was used to balance confounding factors between patients with and those without DM. PSM was based on sex, age, smoking and drinking histories, BMI, hypertension, hyperlipidemia, and hypercholesterolemia. We conducted a one-to-one PSM analysis using the nearest neighbor method with a caliper value of 0.001 to adjust for the imbalance of the aforementioned baseline characteristics between the two groups.

## 3 Results

The baseline patient demographics are shown in Table 1. A total of 4,787 patients with 5,768 IAs, including 2,957 (61.8%) females and 1830 (38.2%) males with a mean age of 58 ± 11 years, were included in this study. The mean BMI was 24.8 ± 5.5 kg/m^2^. Among them, 1765 cases (36.9%) had ruptured aneurysms and 855 cases (11.1%) had multiple aneurysms. A total of 531 (11.1%) patients had diabetes, 2,818 (58.9%) had hypertension, 1,414 (29.5%) had hyperlipidemia, and 311 (6.5%) had hypercholesterolemia.

**Table 1:** Baseline demographic and clinical characteristics of patients with ruptured and unruptured aneurysm.

Variable	All cases (*n* = 4,787)	Unruptured (*n* = 3,022)	Ruptured (*n* = 1765)	Univariate analysis	Multivariate analysis	
				*p*	*p*	OR [95% CI]
Sex (female)	2,957 (61.8)	1,827 (38.2)	1,130 (23.6)	0.014	<0.001	1.39 (1.162–1.666)
Age (year)				<0.001		
≤50	1,142 (23.9)	613 (12.8)	529 (11.1)		Reference	
50–60	1,666 (34.8)	1,067 (22.3)	599 (12.5)		<0.001	0.66 (0.564–0.771)
60–70	1,509 (31.5)	1,039 (21.7)	470 (9.8)		<0.001	0.54 (0.459–0.636)
>70	470 (9.8)	303 (6.3)	167 (3.5)		0.002	0.70 (0.557–0.879)
BMI (kg/m^2^)				0.044		
≤18.5	148 (3.1)	83 (1.7)	65 (1.4)		0.286	1.21 (0.855–1.697)
18.5–24	1874 (39.1)	1,153 (24.1)	721 (15.1)		Reference	
24–28	1,933 (40.4)	1,242 (25.9)	691 (14.4)		0.213	0.92 (0.803–1.050)
>28	832 (17.4)	544 (11.4)	288 (6.0)		0.135	0.88 (0.736–1.042)
Smoking				<0.001		
Never	3,431 (71.7)	2,147 (44.9)	1,284 (26.8)		Reference	
Former	340 (7.1)	250 (5.2)	90 (1.9)		0.042	0.73 (0.545–0.989)
Current	1,016 (21.2)	625 (13.1)	391 (8.2)		0.782	1.03 (0.839–1.262)
Drinking				0.001		
Never	3,959 (82.7)	2,527 (52.8)	1,432 (29.9)		Reference	
Former	134 (2.8)	96 (2.0)	38 (0.8)		0.432	1.18 (0.777–1.803)
Current	694 (14.5)	399 (8.3)	295 (6.2)		<0.001	1.63 (1.321–2.002)
Diabetes	531 (11.1)	406 (8.5)	125 (2.6)	<0.001	<0.001	0.55 (0.444–0.680)
Hypertension	2,818 (58.9)	1,804 (37.7)	1,014 (21.2)	0.128		
Hyperlipidemia	1,414 (29.5)	880 (18.4)	534 (11.2)	0.406		
HyperCHO	311 (6.5)	175 (3.7)	136 (2.8)	0.010	0.016	1.34 (1.056–1.696)

Variables are expressed as number of patients (%), *p* values and OR [95% CI] were derived from comparisons between the ruptured and unruptured aneurysm groups.

OR, odds ratio; 95% CI, 95% confidence interval; BMI, body mass index; CHO, cholesterol.

In ruptured group, there were 1,130(64.0%) females and 635(36.0%) males, with a mean age of 57 ± 11 years and a mean BMI of 24.50 ± 3.64. In total, 481 patients (27.3%) had a history of smoking, 333(18.9%) had drinking, 125(7.1%) had diabetes, 1,014 (57.5%) had hypertension, 534(30.3%) had hyperlipidemia, and 136 (7.7%) had hypercholesterolemia. However; there were 1827(60.5%) females and 1,195(39.5%) males with a mean age of 58 ± 10 years and a mean BMI of 25.00 ± 6.31 in the unruptured group. There were 875 cases of smoking (29.0%), 495 cases of drinking (16.4%), 406 cases of diabetes (13.4%), 1804 cases of hypertension (59.7%), 880 cases of hyperlipidemia (29.1%), and 175 cases of hypercholesterolemia (5.8%) in the unruptured group.

Female sex, current drinking, and hypercholesterolemia were associated with a higher risk of aSAH in univariate and multivariate analyses (*p* < 0.05; Table 1), whereas old age, former smoking, and diabetes were associated with a lower risk of aSAH in univariate and multivariate analyses (*p* < 0.05). The unruptured group consisted of 13.4% diabetes cases (406/3022), and the ruptured group consisted of 7.1% diabetes cases (125/1765) (odds ratio, 0.55; 95% confidence interval, 0.444–0.680) (*p* < 0.001). Other variables, such as BMI, former drinking, current smoking, hypertension, and hyperlipidemia, were not associated with aSAH in the multivariate analysis (*p* > 0.05). Further analysis revealed that DM was still inversely associated with aSAH in both female and male groups (*p* < 0.05).

The admission HbA1c levels were only available in 40.1% cases with DM (213/531). Among them, 42 cases (19.7%) had ruptured aneurysm and 171 cases (80.3%) had unruptured aneurysm. Increased HbA1c was noticed in 40.5% cases (17/42) with ruptured aneurysm and 48.0% cases (82/171) with unruptured aneurysm,respectively. Increased HbA1c (>7.0) was not associated with a lower risk of aSAH in univariate analyses in this study (*p* = 0.384).

In total, 530 patients with DM were successfully matched to 530 patients without DM after PSM. Before PSM, significant differences were found in age, BMI, alcohol consumption, smoking, hypertension, and hyperlipidemia between the patients with and those without DM (Table 2). After PSM, no statistically significant differences were noted in the distribution of these variables. As shown in Table 2, DM was still associated with a lower risk of aSAH after PSM (*p* < 0.001), and the incidence rate of DM (56.2%) in the unruptured group was still higher than that in the ruptured group (35.7%) (odds ratio, 0.24; 95% confidence interval, 0.185–0.313).

**Table 2:** Baseline demographic and clinical characteristics of patients with and without diabetes.

Variable	Before propensity score matching			After propensity score matching		
	Diabetes			Diabetes		
	No	Yes	*p* value	No	Yes	*p* value
No. of patients	4,256	531		530	530	
Female	334 (7.0)	197 (4.1)	0.570	356 (33.6)	334 (31.5)	0.156
Age (year)			<0.001			0.347
≤50	1,095 (22.9)	47 (1.0)		61 (5.8)	47 (4.4)	
50–60	1,480 (30.9)	186 (3.9)		164 (15.5)	186 (17.5)	
60–70	1,293 (27.0)	216 (4.5)		222 (20.9)	216 (20.4)	
>70	388 (8.1)	82 (1.7)		83 (7.8)	81 (7.6)	
BMI (kg/m^2^)			0.001			0.967
≤18.5	140 (2.9)	8 (0.2)		10 (0.9)	8 (0.8)	
18.5–24	1,694 (35.4)	180 (3.8)		181 (17.1)	180 (17.0)	
24–28	1704 (35.6)	229 (4.8)		224 (21.1)	228 (21.5)	
>28	718 (15.0)	114 (2.4)		115 (10.8)	114 (10.8)	
Smoking			0.011			0.297
Never	3,042 (63.5)	389 (8.1)		378 (35.7)	388 (36.6)	
Former	290 (6.1)	50 (1.0)		42 (4.0)	50 (4.7)	
Current	824 (19.3)	92 (1.9)		110 (10.4)	92 (8.7)	
Drinking			0.033			0.054
Never	3,513 (73.4)	446 (9.3)		427 (40.3)	445 (42.0)	
Former	112 (2.3)	22 (0.5)		15 (1.4)	22 (2.1)	
Current	631 (13.2)	63 (1.3)		88 (8.3)	63 (5.9)	
Hypertension	2,402 (50.2)	416 (8.7)	<0.001	422 (39.8)	415 (39.2)	0.598
Hyperlipidemia	1,230 (25.7)	184 (3.8)	0.006	206 (19.4)	183 (17.3)	0.143
HyperCHO	284 (5.9)	27 (0.6)	0.161	33 (3.1)	27 (2.5)	0.425
aSAH	1,640 (34.3)	125 (2.6)	<0.001	298 (28.1)	125 (11.8)	<0.001

Variables are expressed as number of patients (%), *p* values were derived from comparisons between patients with diabetes and patients without diabetes.

BMI, body mass index; CHO, cholesterol; aSAH, aneurysmal subarachnoid hemorrhage.

## 4 Discussion

This case–control study found that the incidence of aSAH was lower among patients with DM than among those without DM; DM was associated with a low risk of aSAH. This inverse association remained significant in our PSM analysis after adjusting for the main confounding factors.

Our result is consistent with a recent literature review study (11), which included 15 case–control studies and three cohort studies with more than 2,000,000 individuals, and indicated that DM reduces the risk of aSAH. A recent population-based cohort study with 421,768 participants also found that the presence of DM is significantly associated with a decreased risk of SAH (10); however, this study could not clearly distinguish SAH cases with or without aneurysm. A recent case–control study of 3,965 patients found an inverse association between them (12). However, these inverse associations are difficult to explain from a pathological perspective. DM is a well-known risk factor for atherosclerosis and can induce atherosclerotic changes and stiffness of cerebral arteries (5). DM is highly correlated with atherosclerotic and stabilized aneurysms based on intraoperative findings (13), and aneurysms with hypertrophied and stiff atherosclerotic walls seem to be more resistant to hemodynamic pressure and are less likely to rupture (13).

However, hyperglycemia can induce vascular endothelial damage and dysfunction and decrease the expression of cerebral tight junction proteins (14). DM can also promote the inflammation process and increase the levels of MMP9 in the arterial walls, all of which can cause aneurysm wall degradation and rupture (15). Clinically, atherosclerotic changes are associated with wall enhancement on magnetic resonance vessel wall imaging, which may indicate rupture-prone aneurysms (16). No association between DM and aneurysm rupture has been reported previously (8). Recent Mendelian randomization studies did not find a causal association between type 2 DMs and aSAH (17); thus, our results should be interpreted with caution. In the real world, patients with DM have a higher risk of dying from other diseases than patients without DM, which reduces the chances of developing aSAH compared with controls. Many patients with diabetes have a higher chance of being evaluated for DM and its complications, such as cerebral infarction and cerebral vascular stenosis, leading to the diagnosis and treatment of cerebral aneurysms before their rupture (8). Patients with DM may modify and maintain a healthy lifestyle (including more exercise, better diet, and less smoking and drinking) and receive medical care to control DM, comorbidities and complications (including hypertension and hyperlipidemia), all of which may lead to a reduced risk of aneurysm rupture (11). Moreover, good and stable glycemic control have been reported to be associated with a reduced risk of IA rupture in patients with DM in previous study (18), and antihyperglycemic agent use was also reported to be associated with a decreased risk of aSAH (19). However, in this study, the exact incidence rate of DM was unknown, and their control group without antihyperglycemic agent use also included patients without diabetes. We could not found an association between blood glucose control status and aneurysms rupture in cases with DM. To determine whether antihyperglycemic agents reduce the risk of aneurysm rupture, a well-designed case–control study is required.

In our study, we found that former smoking but not current smoking was associated with a lower risk of aSAH, and current drinking but not former drinking had a significantly increased risk of aSAH compared with never users (20, 21). Thus, stopping these modifiable risk factors among patients with unruptured IAs is important. Interestingly, hypertension was not associated with aSAH in this study, and a similar result was reported in a previous study (5). This may be because most patients with hypertension undergo antihypertensive treatment and tend to have normal blood pressure after antihypertensive treatment; antihypertensive drugs may further prevent aneurysm rupture through other mechanisms (22). A recent review study including five case–control studies found that hypercholesterolemia is associated with a lower rupture risk of IAs (23). By contrast, in this study, an inverse relationship was observed; hypercholesterolemia increased the risk of aneurysm rupture. A study also found that hypercholesterolemia may increase the risk of SAH particularly in age ≥60 years and female sex (24). No relationship was revealed in other studies (25, 26). Those results should be cautiously explained, because the definition of hypercholesterolemia in other studies includes those cases with lipid-lowering medicaments (23). Statin therapy has been increasingly used not only for lowering blood lipid levels but also for treating cardiovascular and cerebrovascular diseases. Patients with hypercholesterolemia may gain a protective benefit for aneurysm rupture from statin use (23). Further studies with rigorous inclusion and exclusion criteria should be conducted to determine these associations. BMI was not associated with aneurysm rupture in our study. A recent study found that increased BMI is inversely associated with saccular aneurysm rupture in males and patients aged ≥50 years (12). A recent Mendelian randomization study found that a higher BMI increases the risk of both IA and aSAH (17). The association between BMI and aSAH remains uncertain.

### 4.1 Limitations

A notable strength of this study was its large sample size, including patients with ruptured and unruptured aneurysm and patients with diabetes. The main potential confounding factors were also balanced. However, this study had several limitations. First, it was a single-center, retrospective study. A prospective, multicenter, larger study with long-term follow-up is warranted to establish a causal relationship. However, long-term follow-up of patients with rupture-prone aneurysms does not seem ethical. Second, this study did not distinguish between type 1 and type 2 DM; therefore, a different association may exist between DM and aSAH. The diagnosis of DM was not standardized and was based solely on self-report. Due to the impact of ruptured saccular IA and drug interference, the fasting or random plasma glucose level in patients with ruptured IA is unreliable. Third, HbA1c was not routinely measured, and several patients with undiagnosed diabetes may have been missed in this study. Hyperglycemia control status and the use of hypoglycemic drugs, which was not available in all our cases in this study, may also affect our results. Meanwhile, other treatment for patients with diabetes may also reduce the risk for aSAH. The collected information was not all self-reported, especially in cases of aSAH with a poor clinical condition. Fourth, unobserved confounders, such as a family history of aSAH and the use of antihypertensive agents, statins, and aspirin, were not available. Finally, data of patients who failed to reach the hospital were missing. All the aforementioned factors may have led to biased results. This study focused on the risk factors for aSAH, and further research is needed to evaluate the effects of DM on the incidence and growth of IAs.

## 5 Conclusion

In conclusion, patients with aSAH have a lower incidence of DM, however this case-cohort study could not establish a causal relationship. The implications of our findings require careful evaluation. A prospective and large study with long-term follow-up is warranted to establish a causal relationship.

## Data availability statement

The original contributions presented in the study are included in the article/supplementary material, further inquiries can be directed to the corresponding author.

## Ethics statement

The studies involving humans were approved by the ethics committee of Qilu Hospital of Shandong University. The studies were conducted in accordance with the local legislation and institutional requirements. Written informed consent for participation was not required from the participants or the participants’ legal guardians/next of kin in accordance with the national legislation and institutional requirements.

## Author contributions

WZ: Data curation, Formal analysis, Methodology, Writing – original draft, Writing – review & editing, Funding acquisition. KC: Data curation, Writing – original draft, Writing – review & editing. ZS: Data curation, Validation, Writing – review & editing. YX: Data curation, Validation, Writing – review & editing. DZ: Data curation, Writing – review & editing. MZ: Formal analysis, Methodology, Writing – review & editing. YW: Writing – review & editing, Data curation. DW: Validation, Writing – review & editing. WS: Validation, Writing – review & editing, Funding acquisition.
